# A numerical study on the relationship between the doping and performance in P3HT:PCBM organic bulk heterojunction solar cells

**DOI:** 10.1038/s41598-023-29291-8

**Published:** 2023-02-04

**Authors:** Hossein Movla, Afshin Shahalizad, Asghar Asgari

**Affiliations:** 1 Faculty of Physics, University of Tabriz, Tabriz, Iran; 2 Research Institute for Applied Physics and Astronomy (RIAPA), University of Tabriz, Tabriz, Iran; 3 Genoptic LED Inc., 6000 72 Avenue SE, Calgary, AL T2C 5C3 Canada; 4 Photonics Center of Excellence, University of Tabriz, Tabriz, Iran; 5 School of Electrical, Electronic and Computer Engineering, University of Western Australia, Crawley, WA 6009 Australia

**Keywords:** Solar cells, Solar energy and photovoltaic technology

## Abstract

In this study, we perform a simulation analysis to investigate the influence of p-type and n-type doping concentration in BHJ SCs using the drift-diffusion model. Specifically, we investigate the effect of doping on the charge carrier transport and calculate the above-mentioned device parameters. We show that doping the active layer can increase the cell characteristic parameters, that the results are in an excellent agreement with the experimental results previously reported in the literature. We also show that doping causes space charge effects which subsequently lead to redistribution of the internal electric field in the device. Our results reveal that higher doping levels lead to screening the electrical field in the P3HT:PCBM active region. This in turn forces the charge carrier transport to be solely dominated by the diffusion, consequently decreasing the performance of the device. We also show that doping of the active layer to an optimum level can effectively improve the charge transport. Moreover, we show that doping can create an Ohmic contact between the organic and cathode interface. Additionally, the charge carrier concentration profile shows that by increasing the dopant concentration, the $J_{sc}$ can be improved remarkably. Upon doping the active layer, this indicates that illumination can simply reduce the series resistance in the device.

## Introduction

**Figure 1:**
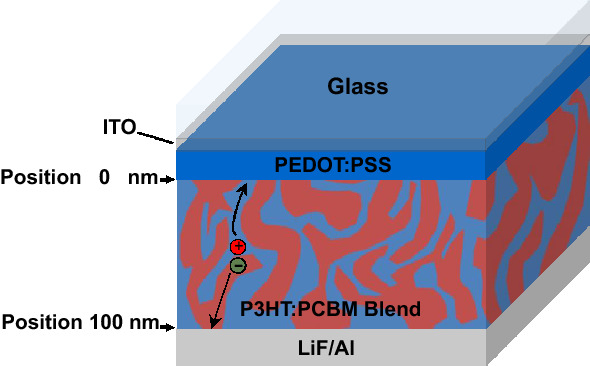
Schematic diagrams of BHJ SC based on P3HT:PCBM blend, and charge carrier transport pathway toward the anode (position 0 nm) and cathode electrodes (position 100 nm).

In recent years, there is a huge effort to the development of efficient, flexible, environmentally stable and lightweight organic bulk heterojunction solar cells (BHJ SCs)^1–4^. These devices are typically made by blending an electron-donor with an electron-acceptor. The functional materials are typically small-molecules or conjugated polymers^5^. Figure 1 shows schematic diagram of a BHJ SC based on poly(3-hexylthiophene) (P3HT) and [6,6]-Phenyl C 61 butyric acid methyl ester (PCBM) with charge transport pathways for the photogenerated charge carrier inside the active region of device (P3HT:PCBM blend). In such an organic donor-acceptor (D-A) blend network, the photogenerated electrons and holes are transported respectively through the acceptor and donor materials^5,6^.P-type and n-type doping of organic semiconductors has proven as one of the most promising techniques for tuning the optoelectronic properties of BHJ SCs^7,8^. In the case of inorganic semiconductors, p-doping is done through incorporating some impurity atoms—possessing one or more missing valence electrons—in the crystalline structure of the semiconductor (i.e., the acceptor). On the other hand, an inorganic semiconductor can be n-doped by incorporating some impurity atoms—possessing one or more extra conduction electrons—into the crystalline structure of the semiconductor (i.e., the donor). Doping alters the electronic properties such as conductivity, internal electrical field, charge carrier mobility, diffusion length etc. However, in organic semiconductors, doping is done by adding charged ions, salts, and surfactants. Unlike inorganic semiconductors, organic material based semiconductors are typically synthesized in a nominally undoped form (i.e., intrinsic)^9^.

In typical inorganic solar cells, doping helps to generate an internal electric field that can separate the electrons and holes in photogenerated excitons and prevent any probable radiative or non-radiative e-h recombination. Also, the doped hole or electron transport layers shown increased conductivities compared to the undoped layers and lead to the better charge transport. Doping organic BHJ SCs typically causes very different effects in the energy band diagrams and electronic characteristics of the functional D-A blend in the active layer^10^. As the experimental results have previously shown that doping can affect the performance of BHJ SCs^11,12^, it can be expected that the characteristic parameters of these devices are highly dependent on the choice of functional materials and dopants^12^. In addition, as a useful step before the device fabrication, numerical models and simulations can allow for predicting and understanding the optoelectronic behaviors of the functional materials in the device structure upon p-type and n-type doping^13–15^. This would also help to study different dopants and find the optimal doping levels to achieve the highest efficiency possible.

Numerical modeling studies have claimed that doping can reduce the efficiency in most cases, and only under some certain conditions, the efficiency can be increased^13^. However, very recent experimental results have demonstrated that molecular or chemical p-type and n-type doping can increase the efficiency of BHJ SCs^8,16,17^. In this study, the effects of doping concentration in the low-mobility regime on the performance of BHJ SCs by numerical simulation was investigated. Indeed, the electrical behaviors of BHJ SCs can be predicted by numerical solving the continuity equations and drift-diffusion equations for charge carriers. In this work, the charge continuity and Poisson equations has been solved by Finite Element Method (FEM) in different p and n-doping density. By considering the device physical parameters and the boundary conditions, device characteristics parameters like the short-circuit current density ($J_{sc}$), open circuit voltage ($V_{oc}$), fill factor (FF), efficiency ($\eta$), and J–V characteristics have calculated. It should be noted in many earlier studies, it was assumed that the recombination of photogenerated charge carriers was bimolecular or Langevin type, but by neglecting other recombination models, it failed to exactly determine generation and recombination processes as well as characteristics parameters of the device^18–20^. In present simulation study, we take into account the bimolecular recombination, charge transferred (CT) recombination, and trap assisted or Shockley-Read-Hall (SRH) recombination models in the charge continuity and drift-diffusion equations (see Supplementary Material for details of the device simulation).

## Models and methods

**Figure 2:**
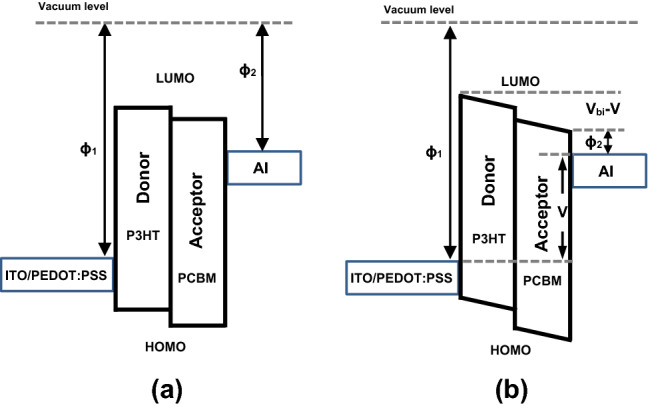
Schematic MIM energy diagram of a BHJ SC in (**a**) flat band and (**b**) under illumination condition.

A simple BHJ SCs is shown by a metal-intrinsic-metal (MIM) scheme^21^. In this scheme, the energy level alignments in the active region comprises the lowest unoccupied molecular orbital (LUMO) of the donor material (P3HT) and the highest occupied molecular orbital (HOMO) of the acceptor material (PCBM). In present device, such a schematic energy level diagram of the different layers consist PEDOT:PSS, P3HT:PCBM and Al is shown in Fig. 2.

In an organic BHJ SC, the hole-transporting polymer is P3HT and the photogenerated electrons are transferred through the electron-transporting PCBM polymeric channels. Recombination in the active region is governed by different mechanisms such as the bimolecular recombination, charge transferred (CT) recombination, and trap assisted or Shockley-Read-Hall (SRH) recombination models (see Supplementary Material for details of the device simulation). For the present device architecture, we have taken these models into account in our numerical simulations.

## Results and discussion

The typical BHJ SC structure shown in Fig. 1 was used in the device simulations. It assumed that the photon absorption in active region generates free e-h pairs. Furthermore, it assumed that doping has no effect on exciton dynamics, and that dopants do not quench excitons. The anode is represented by position 0 nm and the interface between the P3HT:PCBM layer and the cathode is represented by position 100 nm. When an electron and hole pair is formed by photon absorption, charge carriers can be generated in three ways: (1) electrons collect at the cathode, (2) charge carriers recombine, and (3) electrons flow to the anode and accumulate. Photogenerated holes or holes generated by the anode electrode can recombine with electrons. All the parameters used in the numerical simulations are taken from the published reports and are shown in Table 1.

Doping in organic solar cells referred to generate an extra electrical field that separates photogenerated electrons and holes, and also it prevents their recombination^21^. Furthermore doping in certain level increases the conductivity, but after that, it causes decreasing the conductivity and what has yet to be fully understood is how doping affects to the characteristic’s parameters and electrical behavior of BHJ solar cells^22^.

**Table 1:** The parameters that used in numerical simulation.

Parameter	Symbol	Value	Reference
Active layer thickness	*x*	100 nm	^14,15,18,20^
Initial separation of e-h pair	*a*	1.1 nm	^14,15,18,20^
Exciton decay rate	$k_f$	$5\times 10^{6}$ K	^20,21^
Temperature	*T*	300 K	–
Band gap	$E_g$	2.05 eV	^18^
Built-in voltage	$V_b$	0.9 V	^14^
Electron mobility	$\mu _e$	$6\times 10^{-5}$ $\text{cm}^2/\text{Vs}$	^23^
Hole mobility	$\mu _h$	$2\times 10^{-5}$ $\text{cm}^2/\text{Vs}$	^23^
Relative permittivity	$\epsilon (\epsilon _0)$	$3.6\times 10^{-11}$ F/cm	^15^
Effective density of states	$N_c, N_v$	$5\times 10^{17}$ $\text{cm}^{-3}$	^20^
Effective density of states	$N_c, N_v$	$5\times 10^{17}$ $\text{cm}^{-3}$	^20^
Spatial grid	$\Delta x$	1 nm	^20,21^
Time interval temporal grid	$\Delta t$	0.1 s	^21^
Incident sunlight power	$P_{in}$	1000 $\text{Wm}^{-2}$	^14^

**Figure 3:**
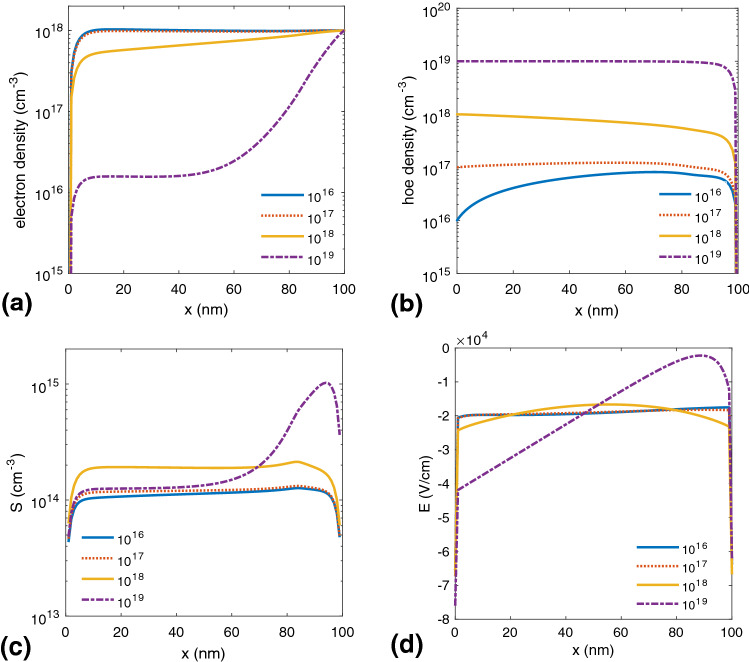
(**a**) Parameters calculated for different p-doping concentrations. Spatial distributions of (**a**) electrons and (**b**) holes, (**c**) exciton density, and (**d**) internal electrical field in terms of the distance from the anode.

Figure 3 shows the spatial distributions of charge carriers, excitons, and internal electric field, respectively.

**Figure 4:**
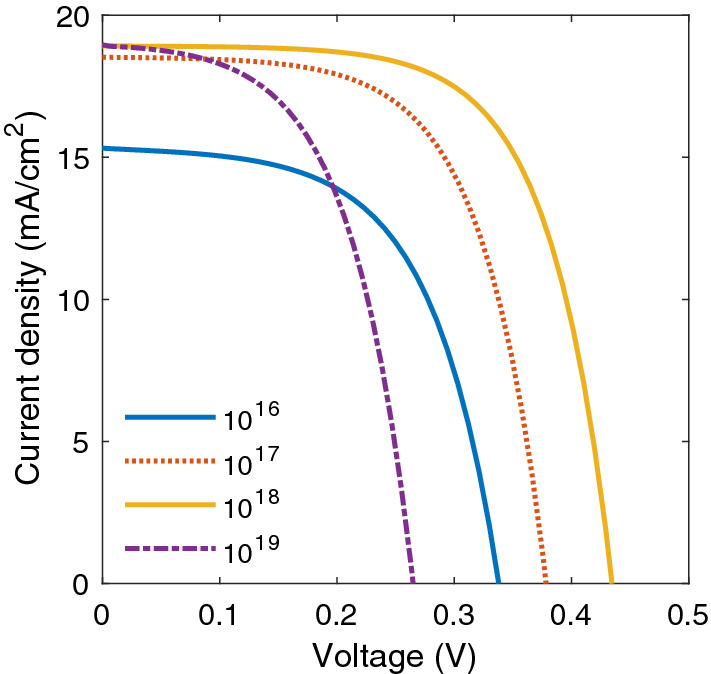
J–V characteristics of a BHJ SC in terms of different p-doping concentrations.

Generally, in undoped organic semiconductor systems, the electron and hole mobilities are not of the same order. P-doping the active layer in a BHJ SC improves the hole density proportional to the p-doping level. The density of excitons, S, also increases for large doping levels due to higher charge carrier generation rates. In this situation, as shown in Fig. 3b, more holes formed, and thus the generated holes tend to more free charge carrier’s generation until $10^{18}$ $\text{cm}^{-3}$ p-doping level. By increasing the doping level above $10^{18}$ $\text{cm}^{-3}$, the exciton density profile changes dramatically, which follows the trend of internal electrical field (see Fig. 3d). Specifically, the exciton density decreases near the middle of the device, but it increases sharply near the cathode. Additionally, in the case of the $10^{18}$ $\text{cm}^{-3}$ doping level, the increase in the electrical field leads to an increased dissociation of the excitons into free charge carriers. In this case, the field-dependent dissociation rate constant in Eq. 4, $k_{diss}$ (see Supplementary Material for details of the device simulation), becomes greater than the exciton decay rate, $k_f$, enhancing the cell characteristic parameters. In BHJ SCs, the holes are much slower than electrons, so they pile up at the interfaces of the organic-organic and organic-metal. In these devices, space-charge effects generally occur due to doping^24,25^ and imbalanced charge transport of charge carriers^21,26,27^. As shown in Fig. 3d, by increasing the p-doping level to more than $10^{18}$ $\text{cm}^{-3}$, due to the space-charge limit, the shape of the internal electrical field changes and reaches a minimum value at the cathode. The space-charge redistributes the internal electric field in the active layer into two different regions: a quasi-neutral region, and a space-charge region. In the quasi-neutral region (right side of Fig. 3d for $10^{19}$ $\text{cm}^{-3}$ doping level), the electrical field in the active layer is screened, and the charge carrier transport proceeds only through diffusion. Consequently, as shown in Fig. 4, charge carriers easily recombine, leading to a decreased device performance. The charge density in the undoped active layer of BHJ SC is near zero, so the energy levels have an almost constant slope, and a nonzero electric field is present throughout the layer^13^. In this area of the active layer, a Schottky barrier is formed because the space charge generates a strong electric field close to the cathode (p doping) and anode (n doping), and in the middle of active layer, due to the high concentration of free charges, the electric field is almost zero, and the energy levels are horizontal.

**Figure 5:**
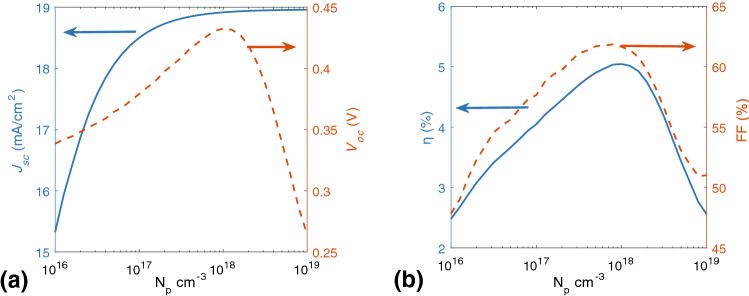
Numerical results calculated for variation of (**a**) $J_{sc}$ and $V_{oc}$ and (**b**) *FF* and $\eta$ as a function of p-dopant concentration.

The reverse side of the space-charge effect is that the internal field is enhanced (left side of Fig. 4 for $10^{19}$ $\text{cm}^{-3}$ p-doping level), which it may even improve the performance of the device. It can be seen that the shapes of J–V characteristics significantly change. As expected from Fig. 3, the space-charge effects play a major role in higher doping levels and decreasing the performance of the device. In the literature, it has been proposed that doping could facilitate the increased interdigitation in polymer chains that leads to higher conductivity and better cell efficiency^28^. However, by increasing the doping level, an unusual decrease in the conductivity is observed^28^. Indeed, as electrical conductivity depends on charge carrier density and mobility, heavy doping of the active layer of BHJ SCs causes the space-charge effect and increasing exciton decay rate, electrical conductivity decreases. It is useful to investigate how p-doping concentration affects the performance of the device. Figure 5a shows $J_{sc}$ and $V_{oc}$ and Fig. 5b shows the *FF* and $\eta$ with respect to p-dopant. As it shown in Fig. 5b, the *FF* increases by p-doping density at certain value, and it drops after reaches to maximum value (62.1 %) at about $N_p=10^{18}$ $\text{cm}^{-3}$ p-doping concentration. The main reason for dropping FF by rising the p-doping level is increasing the recombination rate. Actually the ratio at about $N_p=10^{18}$ $\text{cm}^{-3}$ concentration reach to its maximum value and in lower and higher doping level, the ratio decreases. As it was seen in Fig. 5b, $\eta$ reaches to its peak value, 5.1 %, at somewhat higher doping level ($N_p=10^{18}$ $\text{cm}^{-3}$) and after that, drops about 50% and reaches to the 2.4 %. The results for device characteristics parameters imply that the optimum value for p dopant concentration is about $N_p=10^{18}$, respectively. we figure out the characteristic parameters of study case to understand why there is a decreasing in the observed trends in $\eta$.

As it was shown from Fig. 5a, at high p-doping level, $J_{sc}$ is constant and $V_{oc}$ increases and reaches to its peak value, so in this case $J_{max}$ and $V_{max}$ or J–V characteristic determines $\eta$ drops down. Increasing the doping level in active region leads to extra charge carrier generation and also, controlling the photocarrier transport path, and consequently, it leads to increased efficiency. Numerical results of performance data calculated with different p-doping concentrations have shown in Table 2. It is clear that maximum efficiency occurs in $10^{18}$ $\text{cm}^{-3}$ doping concentration with optimal $V_{oc}$ and *FF*.

**Table 2:** Numerical results of performance data calculated with different p-doping concentrations.

Parameter	$10^{16}$ $\text{cm}^{-3}$	$10^{17}$ $\text{cm}^{-3}$	$10^{18}$ $\text{cm}^{-3}$	$10^{19}$ $\text{cm}^{-3}$
$J_{sc}$ ($\text{mA}/\text{cm}^{2}$)	15.32	18.52	18.92	18.96
$V_{oc}$ (V)	0.34	0.38	0.44	0.26
*FF* (%)	47	60	62	51
$\eta$ (%)	2.42	4.15	5.10	2.55

**Figure 6:**
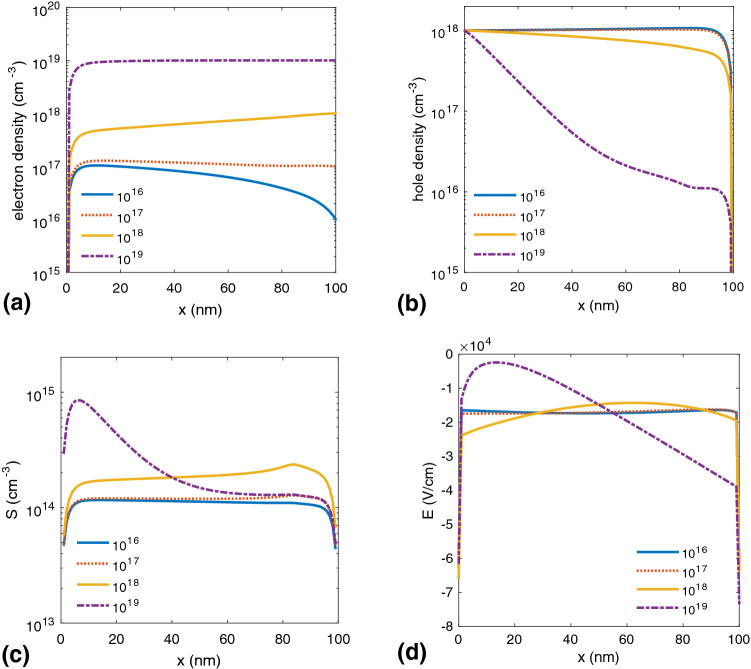
(**a**) Numerical results calculated with different n-doping concentrations for spatial distributions of (**a**) electrons, (**b**) holes, (**c**) exciton density, and (**d**) internal electrical field in terms of distance from the anode.

**Figure 7:**
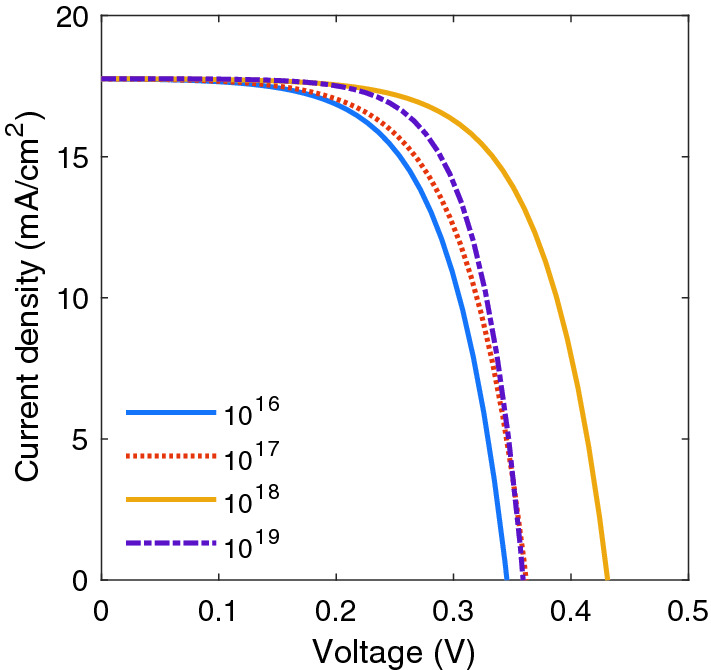
J–V characteristics as p-dopants concentration.

Figure 6 demonstrates spatial distributions of electrons and holes, excitons density and internal electrical field versus distance from anode in various n-doping concentration, respectively. In n-doping condition, same phenomena for space-charge region occurs and only differences is the slop of density of excitons, *S*, changes in reverse side. By increasing the n-doping level, as it has shown seen in Fig. 3a and b, higher concentrations of electrons are observed which causes photogenerated charge careers to reach each other near the anode. As it is shown in Fig. 6d, the electric field is much higher in the n-doped cells, but by increasing the doping, there is an unbalanced electrical field that occurs. As discussed above, increasing the electrical field in the case of $10^{18}$ $\text{cm}^{-3}$ doping density, dissociation rate of charge carriers increase and in $N_d=10^{18}$ $\text{cm}^{-3}$ doping level, field-dependent dissociation rate constants in Eq.4, $k_{diss}$, became higher than exciton decay rate, $k_f$, and it leads to increasing the cell characteristics parameter. Figure 7 demonstrate J–V characteristics of solar cell in different n-doping concentration. By increasing the doping, $J_{sc}$ is relatively constant because of electron and hole mobility imbalance.

**Figure 8:**
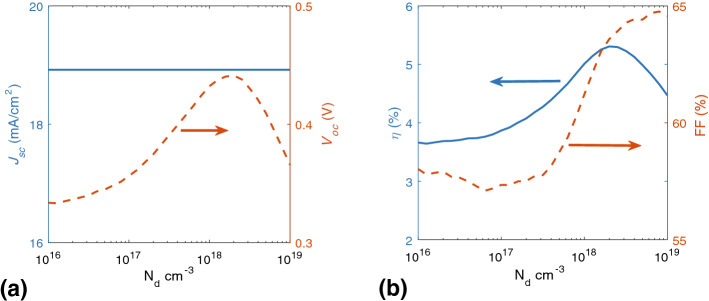
Numerical results calculated for variation of (**a**) $J_{sc}$ and $V_{oc}$ and (**b**) *FF* and $\eta$ as a function of n-dopant concentration.

Figure 8a shows $J_{sc}$ and $V_{oc}$ and Fig. 5b shows the *FF* and $\eta$ with respect to n-dopant. *FF* increases by n-dopant concentration but in all investigated four cases. As it was shown in Fig. 8b, $\eta$ reaches its peak value, 4.54 %, at the $N_d=10^{18}$ $\text{cm}^{-3}$ and after the optimum value for n-doping, it drops. As can be observed, the ideal value for n-doping is about $N_d=10^{18}$ $\text{cm}^{-3}$, and optimum performance data with different n-doping concentrations has shown in Table 3.

Same as the p-doped ones, it is clear that maximum efficiency occurs in $10^{18}$ $\text{cm}^{-3}$ doping concentration with optimal $V_{oc}$ and *FF*. In the n-doping, current density is relatively constant.

**Table 3:** Numerical results of performance data calculated with different n-doping concentrations.

Parameter	$10^{16}$ $\text{cm}^{-3}$	$10^{17}$ $\text{cm}^{-3}$	$10^{18}$ $\text{cm}^{-3}$	$10^{19}$ $\text{cm}^{-3}$
$J_{sc}$ ($\text{mA}/\text{cm}^{2}$)	17.75	17.76	17.77	17.74
$V_{oc}$ (V)	0.34	0.36	0.43	0.36
*FF* (%)	57	57	59	65
$\eta$ (%)	3.50	3.64	4.54	4.10

## Conclusions

In this study, we investigated the effects of the p and n doping concentration on the BHJ SC characteristics parameters based on P3HT:PCBM blend. We perform numerical simulation by drift-diffusion model where FEM has been taken into account in simulations. The J–V characteristics affected by increasing or decreasing dopants from $10^{18}$ $\text{cm}^{-3}$ optimum doping levels, which means that in the p-doping instance, FF reduces with increasing dopants, and hence efficiency decreases. Increasing doping in the active region of studied OSC to the $10^{18}$ $\text{cm}^{-3}$ results in extra charge carrier generation as well as control of the photocarrier transport path, resulting in higher efficiency. Furthermore, simulation of the charge carrier concentration profile reveals that increasing the dopants induces an increase in charge carrier intensity, which leads to an increase in $J_{sc}$. This means that dopants just lowers the solar cell’s series resistance. These findings suggest that doping is quite essential, as it enters through efficiency and has a significant impact on the performance of organic solar cells.

## Supplementary Information

Supplementary Information.

## Data Availability

The datasets used and/or analyzed during the current study available from the corresponding author on reasonable request.
