# Supplementary material for: A numerical study on the relationship between the doping and performance in P3HT:PCBM organic bulk heterojunction solar cells
Source: Sci Rep. 2023 Feb 4;13:2031. doi: 10.1038/s41598-023-29291-8 (PMC9899250; doi:10.1038/s41598-023-29291-8)
Supplement: Supplementary file 1 — Supplementary Information. [file 41598_2023_29291_MOESM1_ESM.pdf]

Supplementary Material

A Numerical Study on the Relationship between the Doping and Performance in P3HT:PCBM Organic Bulk Heterojunction Solar Cells

Hossein Movla [a,b], Afshin Shahalizad [c] and Asghar Asgari [a,b,d,e]

a Faculty of Physics, University of Tabriz, Tabriz, Iran

b Research Institute for Applied Physics and Astronomy, University of Tabriz, Tabriz, Iran

c Genoptic LED Inc., 6000 72 Avenue SE, T2C 5C3, Calgary, Alberta, Canada

d Photonics Center of Excellence, University of Tabriz, Tabriz, Iran

e School of Electrical, Electronic and Computer Engineering, University of Western Australia, Crawley, WA 6009, Australia

In an organic BHJ SC, the hole-transporting polymer is P3HT and the photogenerated electrons are transferred through the electron-transporting PCBM polymeric channels. Recombination in the active layer is governed by different mechanisms such as bimolecular (or Langevin recombination), Shockley-Read-Hall (SRH) recombination, trap assisted, or geminate recombination. We have taken these models into account in our numerical simulations.

A. Bimolecular recombination

Bimolecular (or Langevin recombination), R_0, with the recombination constant (β) and the intrinsic charge carrier density (n_i), which is governed by the electron (n) and hole (p) densities is given by the following equation:

$$R_{lang} = \beta(np - n_i^2) \quad (1)$$

where β is a function of the electron and hole mobilities (μ_n and μ_p):

$$\beta = q\frac{(\mu_n + \mu_p)}{\varepsilon\varepsilon_0} \quad (2)$$

where $\varepsilon\varepsilon_0$ is the permittivity of the active layer P3HT:PCBM blend material, and q is the elementary charge. μ_n and μ_p are electron and hole mobilities, respectively. For undoped functional materials in the active layer, both electrons and holes are transported through the same material

and could move in all directions. Analogous to the previously reported experimental results [1-4], in the present work, we have used unbalanced mobilities of electrons and holes in our calculations.

B. Charge Transferred (CT) recombination

Photogeneration of the free charge carriers has been explained by the Onsager theory [5], and Braun [6] has made an important refinement to this theory by pointing out that a bound e-h pair with binding energy E_B -which acts as a precursor for free charge carriers- has a finite lifetime. E_B is considered as an intermediate state through which the recombination and dissociation of charge carriers are triggered. The charge carriers in an e-h pair can return to their initial states or dissociate into free charge carriers. This charge carrier dissociation is a competition between the separation rate, k_{diss}, and the lossy radiative or non-radiative recombination of charge carriers which undergo transferring through an intermediate state, that is, the charge-transfer (CT) state. In this model the probability of dissociation for a given e-h pair distance x, is given by:

$$p(x,T,E)=\frac{\kappa_{diss}(x,T,E)}{\kappa_{diss}(x,T,E)+\kappa_f(T)} \qquad (3)$$

In this equation, k_{diss} depends on both temperature, T, and electric field strength, E. The decay rate of the bound e-h pair to the ground state, k_f, is used as the fitting parameter. Based on the Onsager theory for a field-dependent dissociation rate constant in the case of weak electrolytes with low mobility [1,5], Braun derives the following expression for k_{diss}:

$$\kappa_{diss}(x,T,F)=\frac{3R}{4\pi a^3}e^{-E_B/k_BT}\frac{J_1(2\sqrt{-2b})}{\sqrt{-2b}} \qquad (4)$$

where $b=e^3F/(8\pi\varepsilon_0\varepsilon_r k_B^2T^2)$, J_1 is the Bessel function of first order ($J_1=1+b+\frac{b^2}{3}+\frac{b^3}{18}+\frac{b^4}{180}+\ldots$), and R is the recombination rate.

As polymer systems in BHJ SCs are subject to disorder, it is reasonable to assume that the e-h pair distance is not constant throughout the system [1]. As a result, it should be integrated over the distribution of separation distances:

$$P(a,T,F)=\int_0^\infty p(x,T,F)\,f(a,x)\,dx \qquad (5)$$

where f(a,x) is a normalized distribution function that is given by [3]:

$$f(a,x) = \frac{4}{\sqrt{\pi}a^3} x^2 e^{-x^2/a^2} \tag{6}$$

This leads to a modification of free charge carrier recombination terms by P that describes the probability of the dissociation of a CT state, and consequently, geminate recombination is determined as [1,4]:

$$R_g = R_{lang}(1-P), \tag{7}$$

where R_g is the geminate recombination, respectively.

C. Shockley-Read-Hall recombination

Another mechanism that can account for losses in organic BHJ SCs is the indirect recombination of charge carriers by traps. To calculate the recombination rate, the trap assisted Shockley-Read-Hall (SRH) with the trap density N_t and the capture coefficient C_t are taken into account [7-9]:

$$R = C_t N_t \frac{np - n_i^2}{n + n_d + p + p_0} \tag{8}$$

which n_d and p_0 are characteristic charge carrier densities. For simplicity, we assume a temperature-independent effective bandgap.

D. The model

The basic equations used in this paper are the Poisson equation:

$$\frac{dE(x)}{dx} = -\frac{q}{\varepsilon_0\, \varepsilon_r}(p(x) - n(x) + N_n - N_p) \tag{9}$$

where E(x) is the applied electric field, N_n and N_p are n-type and p-type doping concentrations, respectively. The current continuity equations are:

$$\frac{d}{dt} n(x) = \frac{1}{e}\frac{d}{dx} J_n(x) + G(x) - R(x)$$

$$\frac{d}{dt} p(x) = \frac{1}{e}\frac{d}{dx} J_p(x) + G(x) - R(x) \tag{10}$$

In these equations, J_n, J_p are the electron and hole current densities, G is the generation rate of free charge carrier result from the separation of e-h excitons, and R is the recombination rate [10]. To calculate the exciton density profile, we can use:

$$\frac{d}{dt} S(x) = G(x) + \frac{1}{4}R(x) - \frac{S(x)}{\tau_s} - k_{diss}S(x) - \gamma_{ns} n(x)S(x) - \gamma_{ps} p(x)S(x) + \frac{1}{e}\frac{d}{dx} J_s(x) \tag{11}$$

where S, τ_s, γ_{ns} and γ_{ps} are the exciton density, lifetime of excitons, and the second order rate constant for the annihilation of excitons into free electrons and holes, respectively [10]. Considering that the total produced charge carriers recombine inside the BHJ, we can ignore the current gradient. We solve these equations via the FEM method by applying the boundary conditions and assuming a typical 100 nm thick active layer. The scheme used to solve the Poisson and continuity equations is based on the work of Gummel [9]. This new potential is then used to update the carrier densities by solving the continuity equations [6,9]. Finally, by calculating the charge continuity equations, it is possible to calculate J_{sc}, V_{oc}, FF, cell efficiency (η), and J-V characteristics for different doping concentrations. Calculating the η is done using the following equation:

$$\eta = \frac{J_{sc} V_{oc} FF}{P_{in}} \tag{12}$$

where P_{in} is the incident optical power.

References

1. Koster L J A, Smits E C P, V. D. Mihailetchi, Blom P W M, 2005 Phys. Rev. B 72 085205
2. Stelzl F F and Wurfel U, 2012 Phys. Rev. B. 86 075315
3. Mihailetchi V D, Koster L J A, Hummelen J C, Blom P W M, 2004 Phys. Rev. Lett. 93 216601
4. Tress W, Leo K, Riede M, 2012 Phy. Rev. B 85 155201
5. Onsager L, 1938 Phys. Rev. 54 554
6. Braun C L, 1984 J. Chem. Phys. 80 4157
7. Sze S M 2005 Physics of Semiconductor Devices (2nd edn.) (NewYork: John Wiley Sons)
8. Movla H, Gorji N E, Sohrabi F, Hosseinpour A, Rezaei M, Babaei H, 2010 Phys. E 42 2353
9. Gummel H K, I 1964 EEE Trans. Elec. Devices 11 455
10. Damian Głowienka, Je˛drzej Szmytkowski, Influence of excitons interaction with charge carriers on photovoltaic parameters in organic solar cells, Chemical Physics 503 (2018) 31–38
